# Comparative Assessment of the Burden of Injury in Sub-Saharan Africa: An Analysis of Estimates From Global Burden of Disease 2021 Study

**DOI:** 10.7759/cureus.73838

**Published:** 2024-11-17

**Authors:** Kenechukwu Igbokwe, Daniel E Onobun, Reginald Ononye, Chijioke Orji, Ethel O Ojo

**Affiliations:** 1 Trauma and Orthopaedics, Gateshead Health NHS Foundation Trust, Gateshead, GBR; 2 Orthopaedics and Trauma, Warwick Hospital, South Warwickshire University NHS Foundation Trust, Warwick, GBR; 3 Urological Surgery, Glan Clwyd Hospital, Bodelwyddan, GBR; 4 Orthopaedics, Liverpool University Hospitals NHS Foundation Trust, Liverpool, GBR

**Keywords:** disability adjusted life years, global burden of disease (gbd), injury, injury prevention, transport injuries

## Abstract

Objectives

Surgical care for traumatic injuries remains a major concern to public health in sub-Saharan Africa. The 2030 Agenda for Sustainable Development recognizes rising inequalities in global health. The objectives of this study were to compare the Global Burden of Disease (GBD) 2021 estimates on injury mortality and disability across sub-Saharan sub-regions by cause-of-injury category.

Methods

We performed a secondary database descriptive study using the GBD 2021 results on injuries in the four sub-Saharan regions. The age-standardized rates of disability-adjusted life years (DALYs) in the sub-regions were assessed over a 10-year duration between 2012 and 2021.

Results

In 2021, the overall burden of injury is estimated to have affected over 42 million people in sub-Saharan Africa. Although 16 percent of this number is in Nigeria, population data suggests that southern sub-Saharan Africa records 104 deaths per 100,000 from injuries [95% uncertainty interval (UI): 96 to 113] which is twice as high compared to western sub-Saharan Africa (50 injury deaths per 100,000; 95% UI 37 to 61). Within the 10-year duration of this study, the injury DALY rates were twice as high in the Southern regions, compared to the Eastern and Western regions. Transport injuries, interpersonal violence, and drowning contributed the most to the burden of injury in sub-Saharan Africa. There is an overall decline in injury-related mortality rates and DALY rates in the region however there is a rise in the rate of police conflicts and executions.

Conclusions

Although these figures are highest globally, gradual improvements in the 10-year duration of this study were identified but these were slow-paced due to rising rates of police conflicts and executions in sub-Saharan Africa. Overall targeted interventions in communities and regional policy-making efforts are essential tools to create a safer region for the teeming young populace in sub-Saharan Africa.

## Introduction

Death and disability from physical force or exposure including interpersonal, self-inflicted or accidental are described as injuries and represent a significant and often overlooked public health challenge worldwide, contributing to 4.48 million deaths globally in 2017 [1]. The burden of injury is particularly dire in low- and middle-income regions, where access to adequate healthcare services is limited, and preventive measures are often under-resourced [2]. Sub-Saharan Africa is one such region where the impact of injuries, including road traffic accidents, interpersonal violence, and occupational hazards, is both substantial and growing [3]. These injuries not only lead to immediate physical harm but also result in long-term disabilities, placing a significant strain on healthcare systems and diminishing the quality of life for those affected [4].

The Global Burden of Disease (GBD) study from 1990 till date has consistently highlighted injuries as a major cause of premature death and disability globally, with sub-Saharan Africa standing out as a region where the injury burden is disproportionately high [5,6]. Injuries in this region pose direct and indirect economic costs, affecting households, communities, and national economies by reducing workforce participation, increasing healthcare expenditure, and exacerbating poverty cycles [5]. For example, road traffic accidents are a leading cause of injury-related deaths, particularly in countries with weak enforcement of traffic laws, limited infrastructure for road safety, and inadequate emergency medical services [3]. The incidence of fatal road traffic accidents has risen steadily over the past few decades in several sub-Saharan African countries, further contributing to the region's high injury burden [4].

Although there has been some progress in strengthening healthcare systems in sub-Saharan Africa vis-à-vis reducing maternal mortality rates, reduced deaths from malaria and increasing community participation, significant disparities remain in the burden of injury between more developed and less developed countries in the region [7]. Despite these disparities in development indices, there are variations in cause-of-injury mortality and disability which complicates policy directions. However, injury prevention efforts are essential to curtail this leading cause of mortality in the predominantly young and active population of sub-Saharan Africa [4]. Its far-reaching consequences for public health and economic development form the backdrop of this study to report the trends of the burden of injuries in sub-Saharan Africa. The Global Burden of Disease 2021 study offers critical data on injury mortality, and disability-adjusted life years (DALYs) in the region, providing valuable insights into regional variations in injury patterns and their underlying causes [5].

## Materials and methods

An overview of GBD 2021 injury estimation techniques is given in this section. Additional information on GBD estimation and analysis can be found in the GBD 2021 summary paper [8]. This study is reported in accordance with the Guidelines for Accurate and Transparent Health Estimates Reporting (GATHER) [9].

Study design

This study is a secondary database systematic analysis of GBD 2021 study estimates for injury in sub-Saharan Africa, the four sub-regions and the 46 countries: in the eastern sub-region - Burundi, Comoros, Djibouti, Eritrea, Ethiopia, Kenya, Madagascar, Malawi, Mozambique, Rwanda, Somalia, South Sudan, Uganda, United Republic of Tanzania, Zambia; in the western sub-region - Benin, Burkina Faso, Cabo Verde, Cameroon, Chad, Côte d'Ivoire, Gambia, Ghana, Guinea, Guinea-Bissau, Liberia, Mali, Mauritania, Niger, Nigeria, Sao Tome and Principe, Senegal, Sierra Leone and Togo; in the central sub-region - Angola, Central African Republic, Congo, Democratic Republic of the Congo, Equatorial Guinea, Gabon; and in the southern sub-region - Botswana, Eswatini, Lesotho, Namibia, South Africa, Zimbabwe.

In GBD 2021, illnesses and injuries were coded using the International Classification of Diseases, Ninth Revision (ICD-9) and the International Statistical Classification of Diseases and Related Health Problems, 10th Revision (ICD-10) to create a comprehensive hierarchy of nested levels. The GBD's four-level standard hierarchical categories were used to group the cause-of-injury categories. Level 1 causes fall under the "Injuries" category (Group III). "Unintentional injury," "transport injury," and "self-harm and interpersonal violence" are the three Level 2 cause-of-injury classifications. Thirty-six injuries that have been previously reported were included in the further grouping of the cause-of-injury categories [8,10].

The Global Health Data Exchange (GHDx) database provides a key systematic and scientific instrument for retrieving annual data on incidence, prevalence, mortality and DALY for injury estimation [8]. These estimates were created on the database through processes that retrieve information from vital registrations, verbal autopsies, mortality surveillance projects, censuses, surveys, and police records. These estimates were visualized along with a 95% uncertainty interval (UI) on GHDx [11]. The 95% UI was determined following several calculations (1000 times), each time sampling from distributions. Uncertainty was as a representation of the 2.5th and the 97.5th percentile of the distribution of 1000 draws of data inputs at each step [8]. Using predefined filter rules on the database, the annual deaths, DALY, and corresponding age-standardized rates in sub-Saharan Africa and the 46 countries of 2012 and 2021 were obtained for all ages and both sexes. All data are computed by direct inquiry and downloaded through the GBD Results Tool [12]. The corresponding age-standardized mortality rate (ASMR) and age-standardized DALY rates (ASDR) are visualized and retrieved from the database by regions and countries.

Data analyses

The results are presented by sub-regions and cause-of-injury categories in tables. World maps were generated to display the distribution and change trends of regional and national injury burden with the GBD compare tool [13]. All calculations and figures were performed and made using EXCEL 2013 (Microsoft® Corp., Redmond, WA, USA). The age-standardized mortality rates (ASMR) and age-standardized DALY rates (ASDR) are reported per 100,000 person-years using the GBD standard population [8]. The age-standardized rates allow comparisons between populations adjusted for differences in age structures. The statistical code used for GBD study estimation is publicly available online [14].

The percent change over the 1990-2019 period is calculated by respectively subtracting the estimates of ASMR and ASDR for the year 2012 from the estimates of ASMR and ASDR for the year 2021 and dividing it by the respective incidence, mortality and DALY estimates of the reference starting-point (i.e., the year 2012). A positive change indicates an increase of the burden resulting from breast cancer during the 30-year study period, whereas a negative change indicates a decrease.

## Results

**Figure 1:**
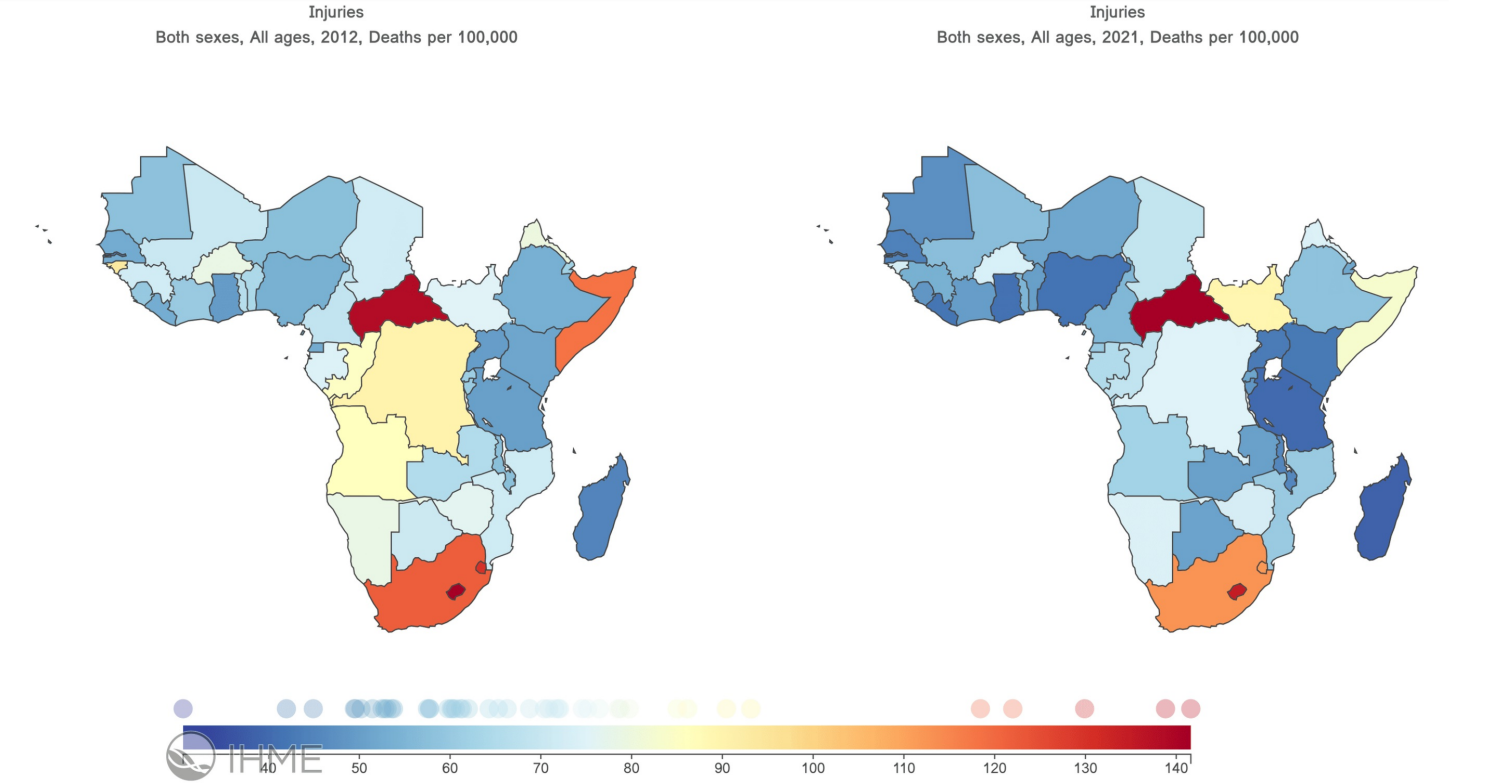
Age standardized mortality rate 2012-2021 in sub-Saharan Africa by countries.

The overall burden of deaths from injuries in sub-Saharan Africa (SSA) is estimated to be rising, estimated at 599,079 (UI: 522,992 - 674,124) in 2012 and rising by 9.3% to 660,954 (556,187 - 770,867) in 2021. In 2021, the injury mortality rate per 100,000 individuals varied between SSA sub-regions. In southern SSA, 104 (95% UI: 96 to 113) individuals per 100,000 died from injuries; twice as high compared to Eastern SSA (injury deaths 54 per 100,000; 95% UI: 47 to 63) and Western SSA (50 injury deaths per 100,000; 95% UI: 37 to 61). Absolute counts show the highest number of deaths attributable to injuries in sub-Saharan Africa were in the western and eastern sub-regions. Road injuries and interpersonal violence were the commonest causes of deaths from injuries in all the sub-regions except in central sub-Saharan Africa where road injuries and self-harm caused the most number of deaths. In 2012 however, road injuries and interpersonal violence were the highest cause of deaths in the eastern [(51743; 95% UI, 46378 - 57032) and (26531; 95% UI, 23910 - 29963)], and southern [(27052; 95% UI, 25751 - 28351) and (24134; 95% UI, 23146 - 25247)], sub-regions while road injuries and drowning were the highest cause of deaths in the western [(59887; 95% UI, 49966 - 69990) and (26411; 95% UI, 21032 - 26411)], and central [(47629; 95% UI, 39803 - 56829) and (12111; 95% UI, 9399 - 15045)] sub-regions. Table 1 shows the number of deaths, per region in sub-Saharan Africa attributable to the overall injuries, and subcategories in 2012 and 2021. Figure 1 shows the ASMR by countries.

**Table 1:** Mortality from injuries 2012–2021 in sub-Saharan Africa. UI - Uncertainty Interval

Cause of Injury	Deaths in 2012, No x 10^3^ (95% UI)				Deaths in 2021, No x 10^3^ (95% UI)			
	East Africa	West	Central	South	East Africa	West	Central	South
Injuries	200.3 (177.2 - 231.3)	221.1 (173.8 - 256.7)	96.8 (81.6 - 114.5)	80.8 (77.5 - 84.4)	229.2 (198.9 - 266.7)	244.3 (180.2 - 296.8)	103.8 (84.5 - 124.6)	83.5 (76.8 - 90.4)
Transport injuries	55.6 (50.4 - 61.2)	62.7 (52.5 - 72.9)	49.1 (41.2 - 58.5)	28.0 (26.7 - 29.4)	62.9 (55.1 - 71.9)	69.7 (55.4 - 84.3)	53.9 (43.1 - 65.4)	29.3 (27.0 - 31.8)
Road injuries	51.7 (46.4 - 57.0)	59.9 (50.0 - 70.0)	47.6 (39.8 - 56.8)	27.1 (25.8 - 28.4)	58.9 (51.3 - 67.0)	66.7 (52.8 - 81.1)	51.9 (41.5 - 63.2)	28.4 (26.1 - 30.8)
Other transport injuries	3.8 (3.0 - 4.7)	2.8 (2.4 - 3.2)	1.5 (1.1 - 2.1)	1.0 (0.9 - 1.1)	4.0 (3.1 - 5.2)	3.0 (2.5 - 3.6)	2.0 (1.4 - 2.6)	0.9 (0.8 - 1.08)
Falls	17.3 (15.0 - 20.1)	19.8 (17.1 - 23.4)	3.9 (3.0 - 5.3)	1.6 (1.4 - 2.0)	21.2 (17.9 - 24.9)	23.4 (19.1 - 27.6)	4.8 (3.4 - 6.8)	1.7 (1.5 - 2.2)
Drowning	17.1 (14.7 - 19.8)	26.4 (21.0 - 32.1)	12.1 (9.4 - 15.0)	3.2 (2.9 - 3.5)	15.4 (12.8 - 18.2)	24.0 (16.8 - 31.2)	9.0 (6.7 - 11.6)	3.3 (2.9 - 3.8)
Fire, heat, and hot substances	13.2 (10.4 - 16.8)	13.8 (6.0 - 18.0)	4.8 (3.6 - 8.2)	3.6 (3.2 - 4.3)	12.9 (9.9 - 17.7)	13.9 (5.5 - 19.4)	4.4 (3.1 - 8.8)	3.6 (3.1 - 4.2)
Poisonings	5.3 (3.9 - 7.2)	4.3 (3.1 - 5.5)	1.6 (1.1 - 2.5)	1.3 (1.0 - 1.5)	5.0 (3.4 - 7.0)	4.3 (2.8 - 5.8)	1.4 (0.9 - 2.4)	1.3 (1.0 - 1.5)
Exposure to mechanical forces	8.0 (5.4 - 13.3)	5.5 (3.9 - 8.7)	2.0 (1.4 - 3.4)	1.8 (1.6 - 2.1)	8.2 (5.5 - 13.0)	5.8 (3.7 - 8.6)	1.9 (1.2 - 3.6)	1.8 (1.5 - 2.1)
Adverse effects of medical treatment	8.4 (5.8 - 17.2)	15.5 (10.5 - 19.4)	2.5 (1.8 - 3.8)	1.2 (1.0 - 1.3)	8.0 (5.4 - 17.0)	15.1 (8.8 - 18.8)	2.2 (1.5 - 3.8)	1.1 (0.9 - 1.3)
Animal contact	7.4 (6.3 - 9.6)	6.9 (4.3 - 8.6)	1.8 (1.3 - 2.9)	1.0 (0.9 - 1.1)	7.5 (6.0 - 10.7)	7.0 (4.0 - 9.7)	1.8 (1.2 - 2.9)	1.0 (0.8 - 1.14)
Foreign body	3.5 (2.1 - 4.8)	8.3 (3.6 - 10.2)	2.5 (1.1 - 3.3)	1.4 (0.8 - 1.7)	3.1 (1.7 - 5.0)	8.5 (3.2 - 11.4)	1.9 (0.8 - 2.6)	1.5 (0.9 - 1.8)
Self-harm	23.2 (20.7 - 26.6)	20.7 (17.0 - 24.0)	8.3 (6.7 - 10.7)	11.6 (10.5 - 12.5)	28.6 (24.4 - 34.4)	25.0 (19.7 - 29.6)	11.0 (8.4 - 15.1)	12.6 (10.9 - 14.3)
Interpersonal violence	26.5 (23.9 - 30.0)	25.1 (20.6 - 30.3)	4.0 (3.3 - 4.7)	24.1 (23.1 - 25.2)	29.8 (25.8 - 35.3)	29.8 (22.2 - 37.8)	4.7 (3.6 - 6.0)	24.6 (22.3 - 26.8)
Exposure to forces of nature	0.3 (0.3 - 0.4)	0.5 (0.5 - 0.6)	0.02 (0.02 - 0.02)	0.02 (0.01 - 0.02)	0.1 (0.1 - 0.1)	0.1 (0.1 - 0.1)	0.08 (0.07 - 0.09)	0.04 (0.04 - 0.05)
Environmental heat and cold exposure	0.9 (0.4 - 1.4)	1.0 (0.2 - 1.4)	0.4 (0.9 - 0.6)	0.6 (0.3 - 1.0)	1.08 (0.05 - 1.71)	1.1 (0.2 - 1.7)	0.5 (0.1 - 0.9)	0.6 (0.3 - 1.01)
Police conflict and executions	0.3 (0.3 - 0.3)	0.4 (0.4 - 0.5)	0.03 (0.03 - 0.03)	0.1 (0.9 - 0.1)	1.11 (1.01 - 1.22)	1.1 (1.0 - 1.2)	0.5 (0.4 - 0.6)	0.2 (0.2 - 0.3)
Conflict and terrorism	5.4 (4.9 - 6.0)	2.7 (2.4 - 3.1)	2.3 (2.1 - 2.5)	0.2 (0.2 - 0.2)	17.3 (10.4 - 28.4)	8.5 (7.5 - 9.7)	4.5 (4.2 - 4.7)	0.04 (0.04 - 0.04)
Other unintentional injuries	7.65 (4.2 - 16.5)	7.4 (4.0 - 11.1)	1.6 (1.0 - 2.3)	1.0 (0.8 - 1.2)	6.91 (3.73 - 14.7)	7.1 (3.3 - 10.2)	1.3 (0.7 - 2.0)	0.9 (0.6 - 1.17)

The injury DALY rate per 100,000 was highest in the southern sub-region (6,094; 95% UI, 5,606 - 6,608), followed by the central sub-region (4,835; 95% UI, 3,924 - 5,831) and then the western sub-region (3,230; 95% UI, 2391 - 3,942). In the central sub-region, drowning (481; 95% UI, 351 - 621) and road injuries (2,338; 95% UI, 1,865 - 2,855) contributed most to the injury DALY rate. However, interpersonal violence and road injuries contributed the highest DALY rates in the southern [(1861; 95% UI, 1688 - 2016) and (1975; 95% UI, 1810 - 2149)], eastern [(474; 95% UI, 411 - 560) and (742; 95% UI, 639 - 839)], and western subregions [(416; 95% UI, 321 - 520) and (786; 95% UI, 615 - 970)]. Trends in DALYs due to injuries between 2012 and 2021 showed a marked increase in disability due to ‘police conflict and executions’ and ‘conflict and terrorism’ across the entire region. This change was highest in central sub-Saharan Africa for police conflict and executions (806%). Central sub-Saharan Africa also reports the highest increase in disability from injuries secondary to exposure to forces of nature (167%). The trends also show a comparable decline in DALY rates from road injuries, drowning and interpersonal violence between 2012 and 2021. Table 2 shows the trends in DALY rates in 2021 and the percentage change in DALYs between 2012 and 2021.

**Table 2:** DALY rates and percent change in DALYs 2012–2021 by cause of injury (Level 3) and by sub-Saharan Africa sub-region with 95% uncertainty interval, 2021 UI - Uncertainty Interval

Cause of Injury	DALY rate x 10^1^ per 100,000 population, 2021 (95% UI)				Percent of change (%) (2012 – 2021)			
	East	West	Central	South	East	West	Central	South
Injuries	333.0 (288.0 - 399.2)	323.0 (239.1 - 394.2)	483.5 (392.4 - 583.1)	609.4 (560.6 - 660.8)	-11.7	-20.6	-22.2	-8.5
Transport injuries	80.2 (69.5 - 91.0)	82.5 (64.9 - 101.2)	242.4 (193.2 - 296.2)	204.6 (187.1 - 222.8)	-12.6	-20.5	-20.2	-6.7
Road injuries	74.2 (63.9 - 83.9)	78.6 (61.5 - 97.0)	233.8 (186.5 - 285.5)	197.5 (181.0 - 214.9)	-12.2	-20.5	-20.7	-6.4
Other transport injuries	6.0 (4.7 - 7.5)	3.9 (3.3 - 4.6)	8.6 (6.3 - 11.4)	7.0 (6.1 - 8.0)	-17.1	-21.7	-2.1	-13.6
Falls	23.7 (20.0 - 27.7)	24.6 (20.5 - 28.8)	19.7 (15.5 - 25.7)	15.8 (13.2 - 19.5)	-5.1	-8.5	-5.9	-4.3
Drowning	24.8 (20.1 - 30.3)	37.3 (25.5 - 49.4)	48.1 (35.1 - 62.1)	26.8 (23.2 - 31.2)	-31.6	-47.7	-46.1	-7.9
Fire, heat, and hot substances	22.7 (17.8 - 29.8)	22.3 (11.0 - 30.1)	21.1 (15.4 - 40.0)	28.7 (24.4 - 33.4)	-24.5	-32.2	-35.3	-14.1
Poisonings	7.2 (4.7 - 10.7)	5.8 (3.9 - 8.0)	6.0 (3.8 - 10.8)	9.3 (7.2 - 11.4)	-29.9	-35.5	-37.0	-12.3
Exposure to mechanical forces	14.7 (10.5 - 22.3)	10.3 (7.5 - 14.0)	10.3 (7.2 - 17.6)	16.0 (13.4 - 18.7)	-17.8	-21.0	-25.4	-15.3
Adverse effects of medical treatment	11.3 (7.6 - 21.1)	21.7 (12.2 - 27.6)	9.1 (6.1 - 13.8)	6.5 (5.1 - 7.5)	-29.6	-39.1	-39.1	-19.2
Animal contact	10.1 (7.9 - 14.5)	9.2 (5.1 - 12.5)	7.4 (5.0 - 12.4)	6.6 (5.5 - 7.7)	-22.8	-32.1	-31.9	-11.6
Foreign body	7.2 (4.7 - 10.9)	15.0 (6.7 - 19.7)	12.3 (6.5 - 16.4)	12.4 (7.9 - 15.0)	-28.1	-29.4	-41.0	-9.5
Self-harm	30.6 (26.1 - 37.2)	22.9 (17.9 - 27.4)	36.5 (27.7 - 50.3)	80.6 (69.7 - 92.4)	-0.8	-6.0	3.5	-3.5
Interpersonal violence	47.4 (41.1 - 56.0)	41.6 (32.1 - 52.0)	26.5 (21.3 - 33.0)	186.1 (168.8 - 201.6)	-10.1	-10.3	-10.0	-9.9
Exposure to forces of nature	0.3 (0.3 - 0.4)	0.3 (0.2 - 0.3)	0.5 (0.4 - 0.5)	0.4 (0.4 - 0.5)	-57.6	-311.2	167.0	52.5
Environmental heat and cold exposure	1.6 (0.8 - 2.3)	1.7 (0.6 - 2.5)	2.1 (0.7 - 3.6)	3.8 (2.0 - 6.6)	-11.9	-15.6	-11.6	-16.3
Police conflict and executions	2.1 (1.9 - 2.3)	1.9 (1.7 - 2.1)	2.8 (2.6 - 3.2)	2.3 (1.7 - 3.5	205.1	45.3	806.1	40.3
Conflict and terrorism	37.0 (23.7 - 59.7)	14.1 (12.3 - 16.3)	30.9 (27.5 - 35.6)	0.8 (0.6 - 1.0	58.1	52.1	20.2	-177.7
Other unintentional injuries	12.1 (7.3 - 23.4)	11.8 (6.5 - 15.9)	7.8 (4.5 - 10.8)	8.8 (6.6 - 10.8)	-27.4	-34.0	-34.8	-23.9

## Discussion

Overall, transport injuries were the highest cause of injury-related deaths in the region, contributing to more than half of the deaths in central Africa and just about a quarter of the overall deaths in other sub-regions. This trend was sustained even after a 10-year interval. Estimates from the World Health Organization state that deaths from transport injuries are highest worldwide and are nearly thrice the deaths in the region compared to their European counterparts [15]. This necessitates the inclusion of injury prevention in targets 3.6 and 11.2 (both related to road safety) of the Sustainable Development Goals [16]. Having been reported as the leading cause of death among people aged 15 and 29 years, death and disability from road injuries present significant drawbacks on their societal participation in various industrial strata and will lead to unexpected loss of productivity with a negative economic impact on the already struggling gross domestic product of many countries in sub-Saharan Africa. Given that many countries in sub-Saharan Africa lack widely available insurance and social protection schemes, expenses are borne by victims and their families [17,18]. This presents grave consequences to the already impoverished families as they experience debts or catastrophic health expenditures due to direct medical costs of accessing care and income loss because of the injury and care access [19].

According to this systematic analysis, the death rates from injury vary widely by sub-region and cause-of-injury category. Despite these variations, we found that age-standardized rates of disability-adjusted life years for all injury categories decreased overall in sub-Saharan Africa between 2012 and 2021. However, this decline was slower in the southern sub-region. This could be attributed to high ASDR from interpersonal violence in the sub-region being highest in sub-Saharan Africa. Further analysis of the data in the southern sub-region showed the highest ASDR resulting from interpersonal violence in sub-Saharan Africa is highest in South Africa, closely followed by Lesotho and then Eswatini in 2012 and 2021. Interpersonal violence in South Africa established in several reports has been recorded as an epidemic accounting for a third of trauma room admissions and also resulting in mortality counts which are seven times the global rate [20,21]. The breakdown of the social fabric is linked to the root causes of violence, many of which are holdovers from the apartheid era. This climate of violence has been exacerbated by gender inequality, family dissolution, high unemployment, corruption and a weak rule of law, income disparity and poverty, and rapid societal change [22,23]. More than half (54%) of all homicides are connected to weapons, making gang violence and easy access to firearms the primary contributing causes. A recent report suggested that a community justice system in certain South African communities often executed by the community vigilantes who are established outside of appropriate law enforcement systems resulted in these high rates and were often associated with higher morbidity for the patient, increased demand for intensive care and depletion of financial resources by the patients’ families [21,24]. The slower decline is therefore not surprising given the huge burden of interpersonal violence in the sub-region.

Although there is a decline in the overall ASMR and ASDR from injuries in sub-Saharan Africa between 2012 and 2021, there is a slow pace of progress which might also be a result of a concomitant marked rise in disability rates resulting from police conflict and executions. Similar studies on injury trends in Europe report the declining ASDR in the continent’s injury rates however with a greater pace [25,26]. Many countries in the region experience high levels of violence, often exacerbated by political instability, economic inequality, and social unrest and this has been compounded by a tripling rise in executions in sub-Saharan Africa [27,28]. These findings highlight the importance of targeted interventions to address specific causes of injury-related deaths and disability in different sub-regions. Despite the importance of targeted interventions, concerted regional efforts might be beneficial in minimizing injuries from conflict and executions. This data is essential for informing evidence-based policy decisions and prioritizing public health interventions aimed at reducing injury-related deaths and disabilities.

GBD 2021's estimates of disease burden by nation and subnational location are useful for describing health inequities within and within subregions, but they are insufficient on their own for planning and establishing public health priorities. For policy considerations, the cost and feasibility of possible interventions to reduce the burden of different diseases are also critical. This study has focussed on sub-regional differences in injury and has not discussed national and sub-national estimates. This study has also treated injuries as an individual factor causing deaths and has not discussed the burden of the co-factors such as alcohol abuse, smoking, and weakened hospital systems. Therefore, it is possible that the multifaceted injury burden has been underestimated.

## Conclusions

Mortality and disability from injuries within this time period continued to improve in the most populous countries in the region. However, significant setbacks in further improvements have been identified in this study. Being the leading cause of death among young people in the region, there should be an urgency to address the predisposing factors to injury at a policy-making level. Future research could be directed at assessing the inequalities in subnational territories as this might influence policy-making efforts in any of the territories.
